# Improving Photocatalytic Performance from Bi_2_WO_6_@MoS_2_/graphene Hybrids via Gradual Charge Transferred Pathway

**DOI:** 10.1038/s41598-017-03911-6

**Published:** 2017-06-16

**Authors:** Ming Liu, Xin Xue, Shansheng Yu, Xiaoyi Wang, Xiaoying Hu, Hongwei Tian, Hong Chen, Weitao Zheng

**Affiliations:** 1 Department of Materials Science and Key Laboratory of Automobile Materials of MOE, Jilin University, Changchun, China; 2 The Second Hospital, Jilin University, Changchun, China; 3 State Key Laboratory of Automotive Simulation and Control, Jilin University, Changchun, China; 4 College of Science, Changchun University, Changchun, China; 5 Key Laboratory of Optical System Advanced Manufacturing Technology, Changchun Institute of Optics, Fine Mechanics and Physics, Chinese Academy of Sciences, Changchun, 130033 China

## Abstract

The charge transfer from the main catalyst to the cocatalyst is a key factor to enhance catalytic activity for photocatalytic nanocomposite materials. In order to enhance the charge transfer between Bi_2_WO_6_ and graphene, we inlet MoS_2_ as a “stepping-stone” into Bi_2_WO_6_ and graphene. Here, we report an effective strategy to synthesize ternary Bi_2_WO_6_@MoS_2_/graphene nanocomposite photocatalyst by a facile two-step hydrothermal method, which is afforded by assembling two cocatalysts, graphene and MoS_2_, into the Bi_2_WO_6_ matrix with a nanoparticle morphology as a visible light harvester. Compared with Bi_2_WO_6_/graphene, Bi_2_WO_6_/MoS_2_ and pure Bi_2_WO_6_, the Bi_2_WO_6_@MoS_2_/graphene ternary composites exhibit superior photocatalytic activity owing to an enhanced charge carrier separation via gradual charge transferred pathway. This work indicates a promising cocatalyst strategy for designing a more efficient graphene based semiconductor photocatalyst toward degradation of organic pollutants.

## Introduction

As an effective strategy in lessening the energy shortage and environment pollution, semiconductor-based photocatalysis has already attracted extensive attention since it is a facile and environmentally friendly way to take advantage of solar energy^1, 2^. At present, various types of semiconductor photocatalysts have been reported such as TiO_2_^3–5^ and ZnO^6–8^ with broad bandgap which absorbs only ultraviolet light which accounts for only about 3~4% of sunlight. Others like CdS^9–11^ and CdSe^12–14^, with narrow band gap which absorbs visible light, whereas contain cadmium element which is a widespread environmental pollutant with high toxicity. Thus, it is extremely necessary to look for a narrow band gap, non-toxic and environmentally-friendly visible-light photocatalysts. Among the various studied photocatalysts, Bi_2_WO_6_ (BWO) is regarded as a prospective candidate owing to its narrow bandgap (2.8 eV)^15^, photo-stability and environmentally-friendly characteristics to be widely used in photocatalytic fields such as degradation of organic pollutants^16^, O_2_ evolution^17^ and reduction of nitric oxide^18^.

However, the photocatalytic performance of BWO is limited by slow electron transfer and fast charge recombination^19^. To date, a variety of strategies have been used to boost the photocatalytic performance of BWO semiconductor photocatalysts, including the textural design of photocatalysts increasing porosity and surface area^20, 21^, metallic and nonmetallic doping^22, 23^, noble-metal loading^24^, as well as metal oxide and metal hydroxide loading^25, 26^. Among the above methods, fabricating heterostructure assembly nanocomposite with graphene has been seen as an effective approach to promoting the efficiency of photocatalysis, which is a hot method of scientists in recent years^27–29^. Not merely because graphene has a unique two-dimensional (2D) structure, extremely high specific surface area and production on a large scale at low cost, but because it has high conductivity and superior electron mobility^30, 31^. Graphene can effectively transfer electrons of the conduction band (CB) of photocatalysis (such as TiO_2_^32^ or CdS^10^) and holes of the valence band (VB) of photocatalyst like BWO^33^. Hence, we conceive a ternary structure, which it could enhance charge transfer if one was inlet as a “stepping-stone” between BWO and graphene to provide a gradual charge transferred pathway.

In particular, 2D MoS_2_ could be an appropriate candidate as a “stepping-stone” between BWO and graphene with suitable VB position. MoS_2_ with the structure similar to graphene has exhibited a good cocatalytic activity due to its excellent property of S atoms on the exposed edges^34^. However, the poor electrical conductivity of MoS_2_ still restricts the overall photocatalytic reaction activity^35^. Recently, 2D MoS_2_/graphene (MG) composite has been adopted as a hybrid cocatalyst which is taken as the development of graphene-based photocatalysts with better photocatalytic performance. Xiang *et al*. prepared a novel composite material consisting of TiO_2_ nanocrystals grown in the presence of a layered MG hybrid as a high-performance photocatalyst for H_2_ evolution via a two-step hydrothermal process^36^. Kolpin *et al*. prepared CdS-graphene-MoS_2_ samples for a highly cooperative solar production of hydrogen gas from water^37^. Guo *et al*. prepared ZCS@MoS_2_/RGO (reduced graphene oxide, RGO) hybrid photocatalyst by a simultaneous reduction reaction which showed the highest photocatalytic H_2_ production activity (2.31 mmol/h) originating from dual charge transfer pathway from excited ZCS to RGO, then to MoS_2_ due to intimate interfacial structure^38^. Yuan *et al*. prepared the hierarchical MoS_2_-graphene/ZnIn_2_S_4_ photocatalyst showing the highest H_2_ evolution rate of 4169 μmol h^−1^ g^−1^ under visible light irradiation due to the effective charge transfer from ZnIn_2_S_4_ to MoS_2_ through graphene^39^. Nimbalkar *et al*. successfully prepared the TiO_2_-RGO/MoS_2_ hybrid composite photocatalysts with high photocatalytic activity over that of the pure TiO_2_ towards the decomposition of methylene blue under sunlight irradiation^40^. The resultant ternary composite exhibited high photocatalytic activity and stability. Zhang *et al*. obtained Bi_2_WO_6_/MoS_2_/RGO heterojunction via hydrothermal process with nanosized interfacial contact by confined space effect and the photocatalytic Cr (VI) reduction performance is observably improved^41^. Thus, it is a feasible scheme to use MoS_2_ as the “stepping-stone” between BWO and graphene to obtain a novel ternary heterojunction Bi_2_WO_6_@MoS_2_/graphene (BWO/MG) composite.

In this study, we designed a novel ternary heterojunction BWO/MG composite photocatalyst by a two-step hydrothermal method, which showed an enhanced charge carrier separation via gradual charge transfer pathway. Compared with Bi_2_WO_6_/graphene (BWO/G), Bi_2_WO_6_/MoS_2_ (BWO/M) and pure BWO, the ternary BWO/MG exhibited superior photocatalytic performance for the degradation of Rhodamine (Rh) B under visible light irradiation, and the degradation rate reached 0.0115 min^−1^ which was 1.5, 2.3 and 9.7 times the degradation rates of BWO/G, BWO/M and pure BWO, respectively. The enhancement of the photocatalytic activity could be ascribed to not only the large specific surface area and a mass of active adsorption sites of MG hybrid cocatalyst, but also the highly-efficient separation and transmission of the photo-induced charge carriers between BWO and MG cocatalyst in the ternary composite. The establishment of BWO/MG ternary hybrids broaden the range of MG hybrid cocatalyst widely used for the preparation of composite photocatalysts and provides a promising method to improve the performance of photocatalysts.

## Results and Discussion

### Structure analysis

**Figure 1:**
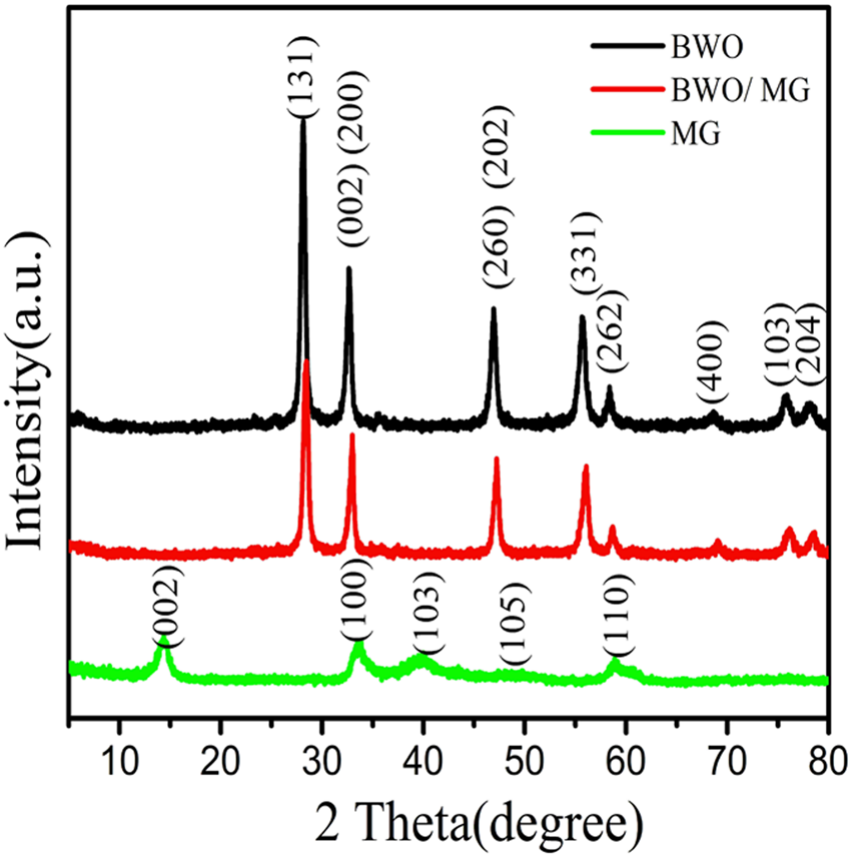
XRD patterns of pristine BWO, BWO/MG and MG composite.

The influences of MG modification on the crystalline structures of BWO nanoparticles were investigated by XRD characterization and corresponding results are shown in Fig. 1. All the peaks for BWO are readily indexed to the orthorhombic phase of BWO. Four strong diffraction peaks at 2θ = 28.09, 32.53, 46.85 and 55.71 can be observed in BWO and BWO/MG samples, which are assigned to (131), (200), (260) and (331) planes of the orthorhombic BWO (JCPDS 73-2020), respectively. It is also seen that the main diffraction peaks of BWO/MG are similar to that of pure BWO. All the diffraction peaks of the hybrids cocatalyst are well indexed according to MoS_2_ phase (as is shown in Fig. S1a). In contrast to the BWO, it can be seen in Fig. S1b that the main diffraction peaks of BWO/xMG (x = 0.5, 1.0, 1.5 and 2.0) are similar to those of pure BWO. No obvious diffraction peak attribute to MoS_2_ or graphene is observed, which suggests that low content and the stacking of the graphene sheets disordered. However, after MG modification the diffraction peaks intensities of BWO crystalline structures are relatively reduced, indicating that the growth of BWO crystals are negatively affected by MG incorporation.

The FTIR spectra of and BWO/MG are shown in Fig. S2. The representative absorption peaks of graphene in BWO/MG including those at 3418.79 cm^−1^, 1620.56 cm^−1^, 1375.96 cm^−1^, and 1076.87 cm^−1^, are O-H stretching vibration, C=O stretching vibration of COOH groups, tertiary C-OH stretching vibration and C-O stretching vibration in good agreement with previous work, respectively^42^. BWO has main absorption peaks between 500 and 1200 cm^−1^, which can be attributed to stretching vibration of Bi-O and W-O, and bending vibration of W-O-W^43^. Obvious decreases in intensity in FTIR spectrum of BWO/MG indicate that the oxygen-containing functional groups in MG are decomposed in the hydrothermal environment. The absorption band appearing at 1620.56 cm^−1^ clearly shows the skeletal vibration of the graphene sheets, indicating the formation of MG in BWO/MG.

**Figure 2:**
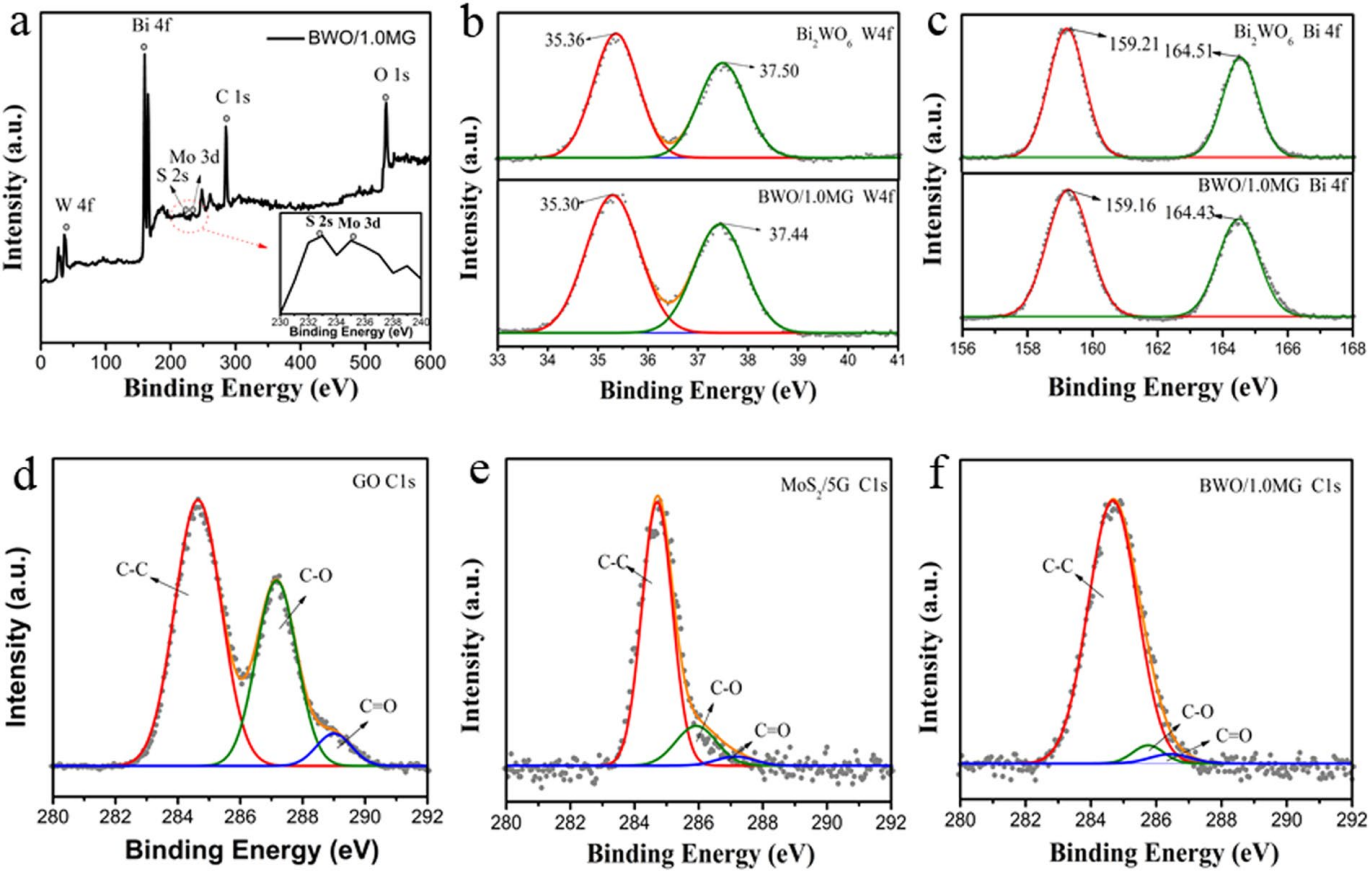
XPS spectra of (**a**) BWO/MG (inset shows a high resolution Mo 3d and S 2 s spectrums in MoS_2_), (**b**) W 4 f and (**c**) Bi 4 f; (**d**) C 1 s spectra of BWO, (**e**) MG and (**f**) BWO/MG.

The interactions between the BWO and MG in the composites are investigated by XPS spectra. The XPS spectrum (Fig. 2a) reveals the peaks of Bi, W, O, Mo, S and C elements. The inset shows a high-resolution Mo 3d spectrum with the peak assigned to Mo^4+^ peak in MoS_2_. Figure 2b–f shows the high-resolution spectra of W 4 f, Bi 4 f and C 1 s, respectively. The peaks with binding energy at 37.50 and 35.36 eV in BWO corresponding to W 4f_5/2_ and W 4f_7/2_, respectively, can be assigned to a W^6+^ oxidation state^18^ (Fig. 2b). Compared with pure BWO, these peaks in BWO/MG were shifted to 37.44 and 35.30 eV, respectively, indicating the presence of a strong interaction between MG and BWO. Note that a similar result was obtained for the binding energies of Bi 4f_5/2_ and Bi 4f_7/2_^18^. It was observed that the peaks of Bi 4f_5/2_ and Bi 4f_7/2_ in BWO/MG were shifted slightly up by 0.05 and 0.08 eV (Fig. 2c), respectively, suggesting chemical bonding between BWO and C elements in MG.

The C 1 s XPS spectra of graphene oxide (GO), MG and BWO/MG are shown in Fig. 2d–f, which can be deconvolved into three peaks corresponding to C-C bond (284.5 eV), C-O in epoxy or hydroxyl forms (287.0 eV), and C=O (289.7 eV), respectively, indicating a high percentage of oxygen-containing functional groups in GO^44^. In contrast, in the XPS spectrum of C1s from the MG and BWO/MG composite, the peak for C-O, and C=O decreases or almost vanishes and the peak for C-C remains almost the same. This indicates that most of the oxygen-containing functional groups are successfully removed. The XPS spectra confirms that the introduced GO has been partially reduced into graphene and -OH groups present on BWO possibly reacted with -COOH groups on the graphene surface through esterification to form O=C-O bonds. The reduction of GO to graphene will dramatically improve the electrical conductivity of the composite, and hence significantly enhancing the photocatalytic activity.

### Morphologies and structure performance

**Figure 3:**
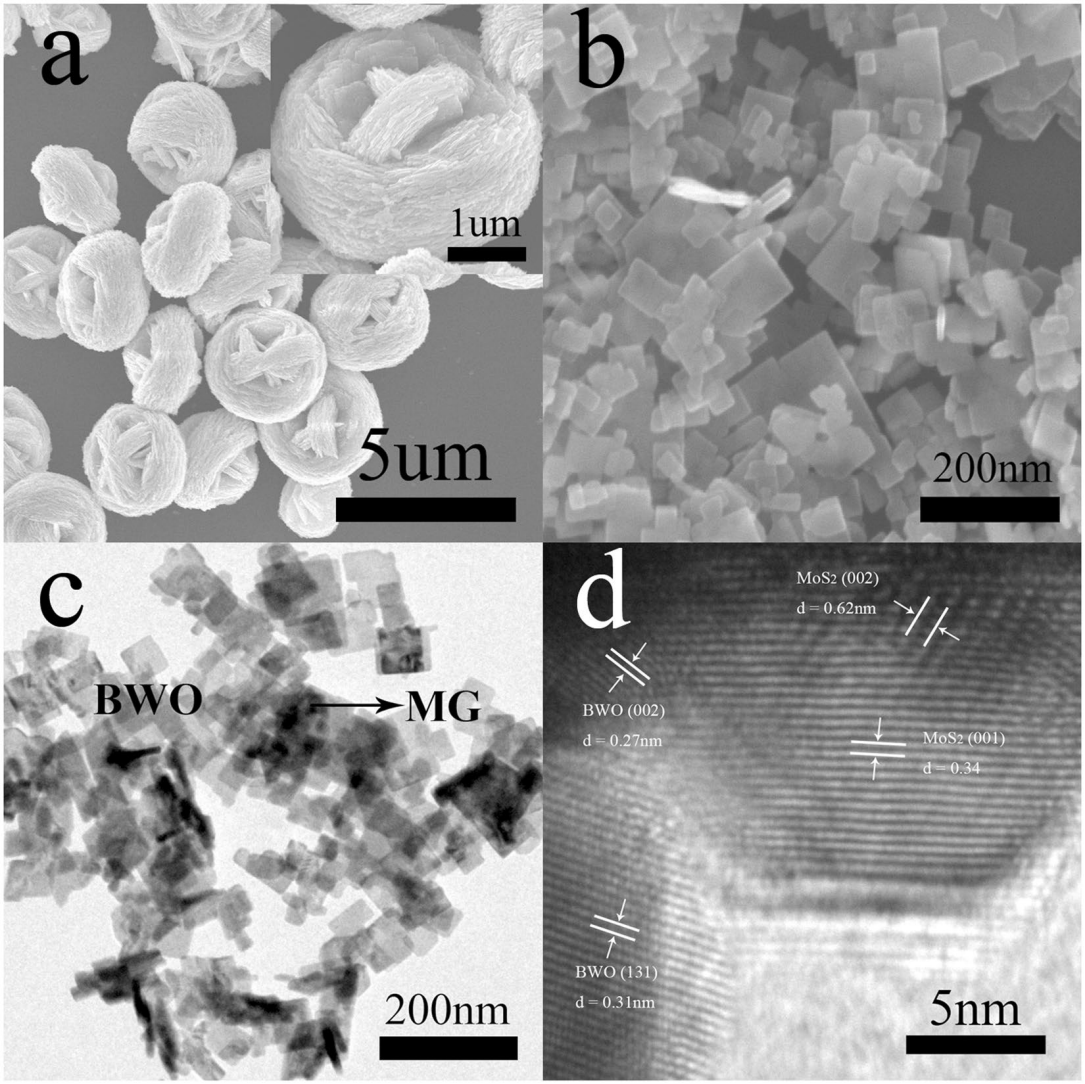
SEM images of (**a**) BWO and (**b**) BWO/MG, (**c**) TEM image of BWO/MG and (**d**) HRTEM image of BWO/MG.

The morphology and microscopic structure of the as-prepared BWO catalyst are characterized using Scanning electron microscopy (SEM). The as-prepared BWO catalyst shows clew-like microspheres structure obtained with an average diameter of 3–4 um as shown in Fig. 3a. The surface of these microspheres was coarse. An amplified SEM image (inset Fig. 3a) indicated that the microsphere was composed of many nanoplates with a lateral size of a few hundred nanometers. Clearly, BWO microspheres had the self-assembled spherical structure from the as-formed nanosheets which assembled from nanoplates, a primary layered crystal structure. The as-prepared MG nanoflowers (as shown in Fig. S3a) are separated using sonication and used as a few layers MG catalyst for the formation of the BWO/MG composite as shown in Fig. 3b. Graphene can be clearly observed in the transmission electron microscopy (TEM) image and high-resolution transmission electron microscopy (HRTEM) image of MG cocatalyst in Fig. S4. After MG modification, the growth of BWO nanosheets are not agglomerated indicating the morphology of BWO crystalline structures are relatively restrained by MG incorporation, which obtained a larger specific surface area to enhance the photocatalytic performance consistent with the results of BET. Furthermore, as evident in Fig. 3b and Fig. S3b–d, the SEM images of the different mass ratio of MG in ternary composites show that the average size of the nanosheets was 50–100 nm, 100–200 nm and 300–500 nm corresponding to BWO/1.0MG, BWO/0.5MG, BWO/1.5MG and BWO/2.0MG, respectively. It can be derived that the concentration of MG reaches 1.0%, the ternary heterojunction BWO/MG composite photocatalyst had the smallest average size of the nanosheets.

In the hydrothermal method, these few layered MoS_2_ and graphene nanosheets are easily decorated on BWO nanoparticles which can be seen in the TEM and HRTEM images. Figure 3c shows TEM image of the resulting BWO/MG composite, in which the layered MG serves as a novel support (Fig. 3d) that is uniformly decorated with BWO. The HRTEM images in Fig. 3d show the structure of the BWO/MG. The lattice fringes of individual BWO with a d spacing of 0.27 nm and 0.31 nm can be assigned to the (002) and (131) lattice planes of BWO in BWO/MG. Notably, Fig. 3d shows that the MG composite has a layered structure with interlayer spacing of ca. 0.62 nm and 0.34 nm, which corresponds to the (002) and (001) planes of hexagonal MoS_2_, respectively. We can see the different-colur elements in the mapping images for Bi, W, O, Mo, S and C elements in Fig. S5a–f based on the selected region in Fig. S5g, indicating that MG nanoparticles were successfully deposited on the surface of BWO/MG. Therefore, a close neighborhood of BWO, MoS_2_, and graphene components achieved by the hydrothermal processing is believed to favor the transfer of photogenerated electrons from BWO to MG sheets, thus enhancing the charge separation and photocatalytic efficiency.

### Photocatalytic study

**Figure 4:**
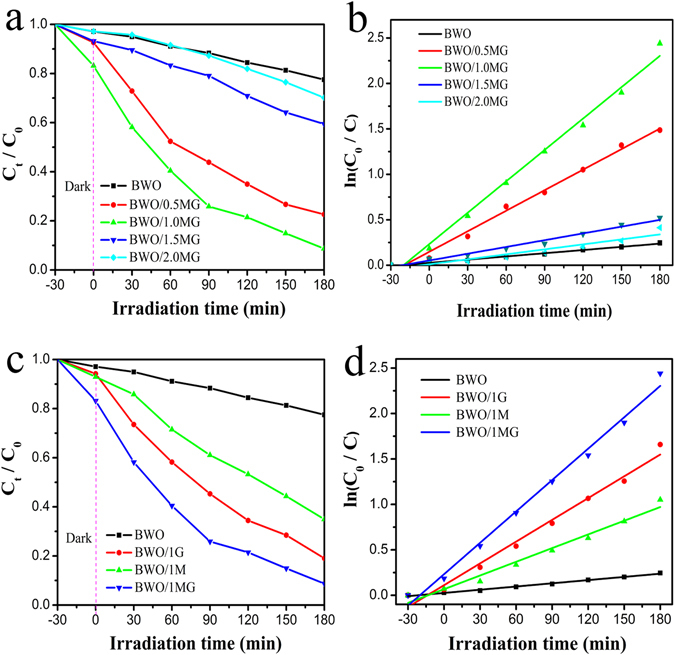
Photocatalytic degradation of RhB over (**c**) BWO, BWO/1 G, BWO/1 M and (**a**) different mass ratio of BWO/MG composites under visible light irradiation (λ > 400 nm). (**a**,**c**) The photodegradation plots and (**b**,**d**) the apparent reaction rate constants k for the photodegradation of RhB. (**c**) the composite photocatalysts containing 1% graphene, MoS_2_ and MG, respectively).

The photocatalytic activity of all the prepared samples was evaluated using Rh B degradation as shown in Fig. 4. Before illumination, the suspensions reached the adsorption-desorption equilibrium after stirring in dark for 30 minutes, as shown in Fig. S6. Under visible light irradiation all samples energy bands are activated through the generation of electron-hole pairs for redox reactions. It is clear that the BWO/MG ternary composite exhibits an excellent photocatalytic activity better than the pure BWO, BWO/1 M and BWO/1 G (Fig. 4c). To investigate the effect of the amount of the MG hybrid cocatalyst (95% MoS_2_ and 5.0% graphene, which photocatalytic activity was best as shown in Table S1) on the photodegradation activity, a series of the BWO/MG composites with different amounts of hybrid cocatalyst (denoted as 99.5BWO/0.5MG, 99.0BWO/1.0MG, 98.5BWO/1.5MG, and 98.0BWO/2.0MG) were examined in comparison to pure BWO (denoted as 100BWO/0MG), as shown in Fig. 4a. The ternary heterojunction of 1.0% MG hybrid and 99.0% BWO showed a highest photodegradation rate. We believe that the observed catalytic activity comes from the interfacial holes transferred to the MoS_2_ surface active sites, the graphene large specific surface area and the simultaneous electrons accumulating at the CB edge of BWO. These transferred electrons on the MoS_2_ active sites and the accumulated holes in the BWO VB edge are mainly participating in degradation of Rh B molecules.

The kinetics of the photodegradation of BWO and different mass ratio of BWO/MG composites are fitted to a pseudo-first order reaction ln(C_0_/C) = k × t, where k, C_0_ and C are the apparent rate constant, initial Rh B concentration and Rh B concentration after a certain time, respectively. The unit for the apparent rate constant is 30 min^−1^. The apparent rate constant of catalysts is shown in Fig. 4b. The value of k was 0.00118, 0.00753, 0.01157, 0.00248 and 0.00183 corresponding to the apparent rate constants of BWO, BWO/0.5MG, BWO/1.0MG, BWO/1.5MG and BWO/2.0MG, respectively. It can be seen that the concentration of MG reaches 1.0%, the ternary heterojunction BWO/MG composite photocatalyst leads to the highest rate (0.0115) of photodegradation which is 1.5, 2.3 and 9.7 times higher than that of BWO/1 G (0.00799), BWO/1 M (0.00502) and pure BWO, respectively (Fig. 4d). As shown in Fig. S7, the degradation efficiency shows a slight decrease after three recycling runs, which directly certify the stability of BWO/MG in the Rh B photodegradation process.

The photocatalytic performance of BWO, BWO/1 G, BWO/1 M and BWO/1MG (contains 95% of MoS_2_ and 5% of graphene in the cocatalyst MG) in aqueous contaminant can also be evaluated by TOC removal. The change of TOC concentration reflected the mineralization degree of Rh B dye in Fig. S8. The TOC removal contents of BWO, BWO/1 G, BWO/1 M and BWO/1MG, respectively, are 32.77%, 34.73%, 47.55% and 77.31% under the visible light irradiation for 180 min. The ternary BWO/1MG catalyst shows the highest TOC removal rate of Rh B among the tested samples, which basically coincides with the result of the degradation rate. This phenomenon suggests that Rh B molecules are most likely mineralized into inorganic molecules.

### Mechanism on enhancement of photocatalytic activity

As is well known to all, the photocatalytic activity of semiconductor photocatalysts is intrinsically governed by three processes: light absorption, charge separation and migration, and surface redox potential. In the following, the mechanisms for the enhanced photocatalytic performance of BWO/MG are explored individually based on the above three processes.

**Figure 5:**
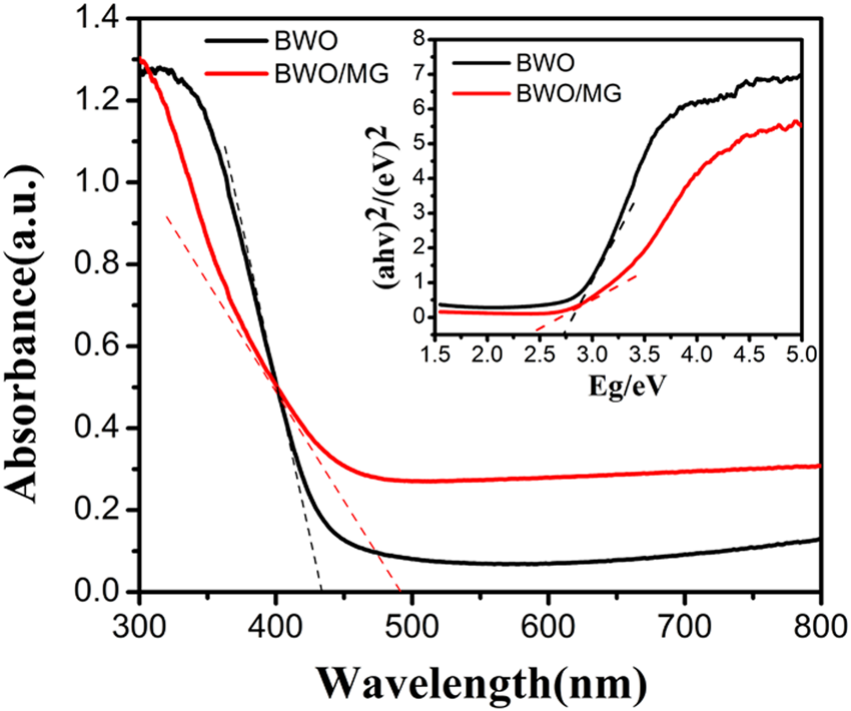
UV-vis diffuse reflectance spectra (DRS) for BWO and BWO/MG (inset presents the corresponding plots of transformed Kubelka−Munk vs energy of light).

The UV-vis DRS of BWO and BWO/MG are compared in Fig. 5. All catalysts exhibit corresponding photo-response in visible light regions due to the intrinsic band-gap. Compared with pure BWO, the BWO/MG nanocomposite shows an enhanced visible light absorption. The band gaps of BWO and BWO/MG were assigned as 2.82 and 2.43 eV, respectively, according to the onset of the absorption edge. As can be expected from absorption spectra, the BWO/MG sample exhibits a narrower band gap and higher absorption intensity than that of pure BWO due to the formation of surface defects^43^. However, the narrower band gap of BWO/MG could be ascribed to MG which has been reported to decrease the band gap of TiO_2_^36^ and CdS^45^. It is generally accepted that the decrease in band gap may decrease the redox potentials of a semiconductor^20^. And reducing the recombination of electrons and holes makes BWO/MG samples show the optimal photocatalytic activity.

Moreover, graphene and MoS_2_ can hinder the recombination of photogenerated electron-hole pairs by rapidly transferring electrons, thus improving the photocatalytic property. The low recombination ratio can be determined by the fluorescence spectra. The higher the fluorescence intensity, the greater probability the recombination of electron-hole pairs. Figure S9 demonstrates the PL spectra for pure BWO and BWO/MG samples under 514 nm excitation wavelengths. The samples present a strong emission peak around 679 nm in Fig. S9, owing to the intrinsic luminescence of samples. It is found that the PL intensity of BWO/MG samples are lower than that of pure BWO, which clearly indicates the inhibition of the recombination of excited electrons and holes in BWO/MG catalysts^41, 43^.

**Figure 6:**
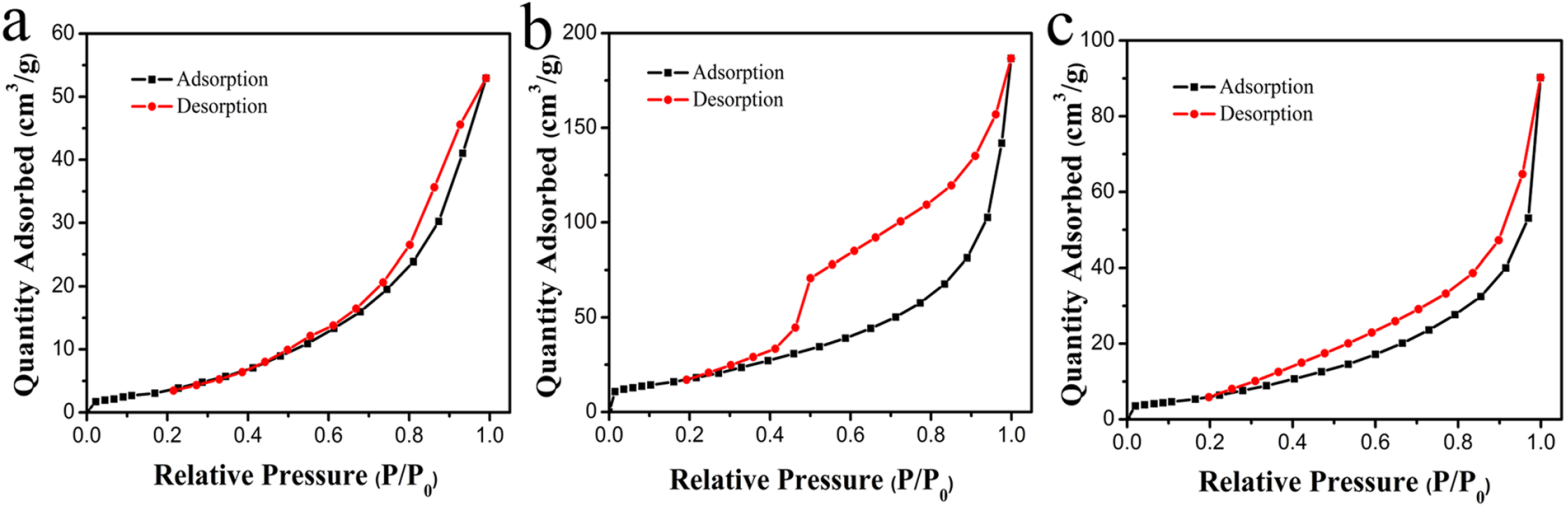
N_2_ adsorption/desorption isotherms of (**a**) pure BWO, (**b**) MG and (**c**) BWO/MG nanoparticles.

The nitrogen adsorption/desorption isotherms of pure BWO, MG and BWO/MG are shown in Fig. 6a,b and c, respectively. The isotherms of Fig. 6a,c reveal a type IV isotherm with a H4 hysteresis loop, suggesting the slit-shaped pores produced by the layered structure. The Fig. 6b displays a type II isotherm with a H2 hysteresis loop, indicating the existence of little micropore stemming from nanoparticles. As revealed by SEM observation, the slit-shaped pores are ascribed to the spaces between the intercrossed MG nanoflowers with a large surface area (57.64 m^2^/g). Based on BET calculation, the surface area of BWO/MG is 18.78 m^2^/g much larger than that of pure BWO (11.54 m^2^/g). These results show that importing MG could increase the surface area of composite, leading to strong adsorption for Rh B.

For attesting to the favorable effect of the grphene and MoS_2_ in BWO/MG toward the light harvesting and transfer of photoexcited electron-hole pairs, photoelectrochemical analysis was performed under visible light. As shown in Fig. S10a, the transient photocurrent responses of BWO, BWO/G, BWO/M and BWO/MG photoelectrodes were still reproducible after several on-off cycles of regular irradiation of the visible light. The BWO/MG photo-electrode had the markedly highest photocurrent responses compared to BWO, BWO/G and BWO/M, which hints at the more efficient transmission and the longer-time separation for the photogenerated carries. The better conductivity of BWO/MG can be confirmed by the Electrochemical impedance spectroscopy (Fig. S10b). BWO/MG electrode has a smaller frequency semicircle compared with BWO, BWO/G and BWO/M electrodes, which meanings the resistance of BWO/MG electrode is lower than others, would best facilitate the migration of the photoexcited carriers, and therefore, the photocatalytic efficiency could be enhanced^46^.

**Figure 7:**
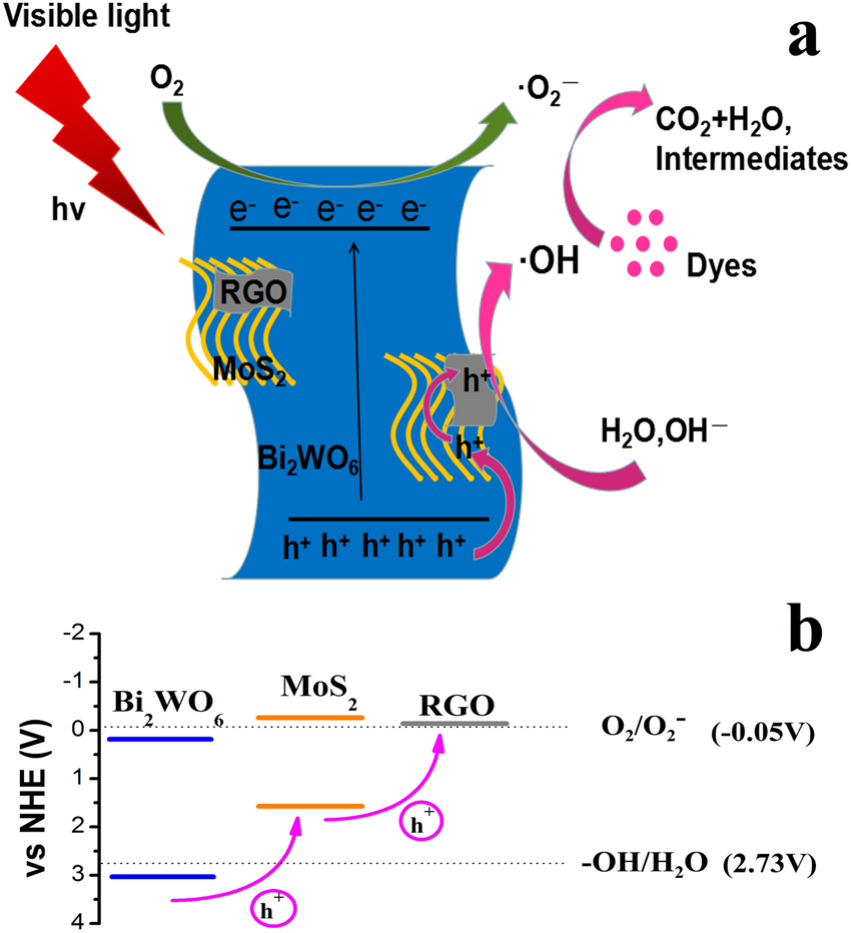
(**a**) Schematic diagram for photocatalytic mechanism of BWO/MG hybrids; (**b**) band structure, RGO, CB and VB levels of MoS_2_ and BWO.

As discussed above, MG plays a critical role in enhancing the photocatalytic activity of BWO/MG composites. As shown in Fig. 7a, we proposed a possible photocatalytic mechanism of BWO/MG ternary hybrids for degradation of organic pollutants. The VB XPS spectra and schematic illustration of the density of states of BWO, MoS_2_ and BWO/MG is shown in Fig. S11. It’s worth noting that both MoS_2_ and BWO can be simultaneously excited to generate electron-hole pairs under visible light irradiation. According to the result of Table 1, 2 the photo-generated holes of MG VB could degrade the organic pollutants directly or react with water to form ·OH radical that further decompose organic pollutants. Thus, after light excitation, the photoexcited electrons in the VB of BWO and MoS_2_ would easily transfer into the CB with leaving holes (h^+^) in the VB. Additionally, the Rh B molecules can also be photoexcited under visible light irradiation. Since the CB level of BWO (ca. 0.23 V vs. NHE) is lower than E^0^ (Rh B*/Rh B^.+^) (−1.09 V)^29, 47^, the photoexcited Rh B* radicals adsorbed on the surface of photocatalyst would also transfer electrons to the CB of BWO and MoS_2_. The photoexcited electrons on the CB of MoS_2_ and graphene both can transfer to the CB of BWO. These photogenerated electrons on the CB of samples can reaction with O_2_ to produce ·O_2_^−^. Meanwhile, graphene and MoS_2_ can capture these photoexcited holes and quicken the mobility hindering the recombination of photogenerated electron-hole pairs. Then these present electrons and holes can emigrate to BWO surface easily and react with the adsorbed molecules directly to produce many strong oxidants including hydroxyl radical (·OH) and ·O_2_^−^ radicals. These oxidative species play crucial roles in the oxidative degradation of organics. At the end of photocatalysis, the BWO/MG hybrids destroy the dye molecules into little pieces, CO_2_, H_2_O and other intermediates. In addition, we plot the band structure, RGO^48^, CB and VB levels of MoS_2_^49^ and BWO, respectively, shown in Fig. 7b. According to the energy band structure, the photogenerated holes on BWO could transfer to RGO. However, MoS_2_ as a “stepping-stone” between BWO and RGO could make photo-generated holes transport from the VB of BWO to the VB of MoS_2_, then to RGO forming a gradual charge transferred pathway with an easier and faster holes transition. Thus, in ternary hybrid composite the photo-generated charge recombination could be effectively suppressed, enhancing the photocatalytic activity.

This indicates that because of a notable synergetic effect between MoS_2_ nanosheets and graphene, the composite cocatalyst has several advantages, including suppression of charge recombination, improvement of interfacial charge transfer, and an increase in the number of active adsorption sites and photocatalytic reaction centers. Notably, the aforementioned gradual charge transferred pathway in which photogenerated holes in the VB of BWO are transferred to improve the separation of the photogenerated electron-hole pairs, effectively prolonging the lifetime of the charge carriers, enlarging the reaction space, and consequently enhancing the photocatalytic activity for photodegradation. The experimental results discussed in this work highlight the synergetic effect of MoS_2_ and graphene as cocatalysts that improve the degradation of Rh B activity of BWO under visible light irradiation. Additionally, this study demonstrates that an effective designed method to fabricate a ternary or multinary photocatalysts via inletting “stepping-stone” into binary photocatalysts, which is a valuable indication for further development of related graphene-based photocatalysts composite materials in photodegradation.

## Conclusion

In summary, we designed a novel ternary heterojunction BWO/MG composite photocatalyst via a two-step hydrothermal method with an enhanced charge carrier separation via gradual charge transfer pathway. MoS_2_ was used as a “stepping-stone” to enhance the holes transition between BWO and graphene. Most importantly, superior photocatalytic activity and stability were exhibited in the ternary composites. The BWO/MG photocatalysts with 1.0% MG cocatalyst showed highest photodegradation activity with a high rate (0.0115 min^−1^) which is almost 1.5, 2.3 and 9.7 times degradation rate of BWO/G, BWO/M and pure BWO, respectively, for the degradation of Rh B under visible light irradiation. It is believed that the positive synergetic effect between the MoS_2_ and graphene sheets as the components of cocatalyst on the photodegradation activity can efficiently suppress charge recombination, improve interfacial charge transfer, and provide a greater number of active adsorption sites and photocatalytic reaction centers. The establishment of BWO/MG ternary hybrid, an inexpensive and environmental visible-light photocatalyst, broadens the range of MG hybrid cocatalyst widely used for the preparation of composite photocatalysts and provides a prospective means to enhance the performance of photocatalysts.

## Methods

### Material

Sodium molybdate dihydrate (Na_2_MoO_4_·2H_2_O) was purchased from Tianjin Fengchuan Chemical Reagent Company (Tianjin, China). Sodium nitrate (NaNO_3_) and sodium tungstate dihydrate (NaWO_4_·2H_2_O) were obtained from Tianjin Guangfu FINE Chemical Works (Tianjin, China). Sulfuric acid (H_2_SO_4_), hydrochloric acid (HCl), ethanol (C_2_H_6_O), hydrogen peroxide (H_2_O_2_) weight ratio 30% and potassium permanganate (KMnO_4_) were provided by Beijing Chemical Works (Beijing, China). Ethylene glycol, thiourea and bismuth nitrate pentahydrate (Bi(NO_3_)_3_·5H_2_O) were supplied by Sinopharm chemical reagent (Shanghai, China). Graphite powder was purchased from Alfa Aesar China (Tianjin, China). All materials were used as received without further purification. Deionized (DI) water used in the preparation was from local sources.

### Synthesis of graphene oxide

Graphite oxide (GO) was prepared by a modified Hummers method using natural graphite fakes as the starting material. In a typical experiment, 1 g graphite powder and 1 g NaNO_3_ were added to 33 mL concentrated H_2_SO_4_ in a 5 °C ice-bath. Then, 6 g ground KMnO_4_ was slowly added while stirring. Then, the mixture was stirred for 90 min at 35 °C, with slowly adding 40 mL distilled water to the mixture. The obtained mixture was stirred for a further 35 min at 90 °C. In the end, 100 mL distilled water was added to the mixture and 30 wt% H_2_O_2_ dropped gradually into it until the mixture turned bright yellow. Then, the mixture was washed with 30% aqueous HCl to get rid of the metal ions, followed by washing with distilled water to effectively remove any remaining metal ions and acids. Finally, the resultant mixture was put into a vacuum drying oven at 60 °C. After 24 h, the brown exfoliated graphite oxide was obtained.

### Synthesis of MoS_2_/grapheneh

The MoS_2_/graphene nanocomposite was synthesized by a one-step hydrothermal reaction of Na_2_MoO_4_·2H_2_O and thiourea in aqueous solution containing exfoliated GO. All of the reagents were of analytical grade and were used without further purification. 135 mg of asprepared GO powders were dispersed into 30 mL of DI water and ultrasonicated until clear yellow-brown suspensions were acquired. After that, a mixture of 1 mmol of Na_2_MoO_4_·2H_2_O and 5 mmol of thiourea was dissolved into the above suspensions and stirred for 30 min. Then the mixture solution was transferred into a 100 mL Teflon-lined stainless steel autoclave, sealed tightly, and heated at an oven at 210 °C for 24 hours. After cooled naturally, the black precipitates were gathered by centrifugation, washed with DI water and anhydrous ethanol in sequence, and finally dried at an oven at 80 °C for 12 hours to obtain as-prepared MoS_2_/graphene powder. With the same method as above, different amounts of GO (according to the weight ratio of GO in composite of 0%, 2%, 5%, 8% and 100%, respectively) were added into the starting material solution.

### Synthesis of Bi_2_WO_6_ and Bi_2_WO_6_@MoS_2_/graphene composites

The nanocomposites were prepared adopting a hydrothermal method. In a typical procedure, 2.28 mmol of NaWO_4_·2H_2_O which was dispersed into 20 mL of DI water and 4.56 mmol Bi(NO_3_)_3_·5H_2_O which was dispersed into 20 mL of ethylene glycol were mixed and stirred for 2 hours to prepare a homogeneous solution. Then 8 mg as-prepared MG powder was dispersed into 10 mL of DI water and 20 mL of ethanol mixture solution to obtain suspensions. The suspensions were added into above homogeneous solution tardily and stirred for 2 hours at room temperature. After that. the solution was transferred to an autoclave for hydrothermal treatment at 180 °C for 10 hours. The autoclave was naturally cooled down to room temperature and washed repeatedly with DI water and ethanol, then dried at 80 °C for 12 hours to obtain BWO/MG composites. As a control, free Bi_2_WO_6_ and other samples were also synthesized using the same procedure under the same reaction conditions without adding GO and varying the 0.5, 1.0, 1.5, 2.0 wt%, respectively, concentration of the MG composites.

### Characterization

The as-prepared catalysts were examined with an X-ray diffractometer (XRD, a Bruker D8) with Cu Ka radiation. Scanning electron microscopy (SEM) images were performed using a field emission scanning electron microscope (FESEM, Hitachi, SU8010). Transmission electron microscopy (TEM) and high-resolution transmission electron microscopy (HRTEM) images were obtained by a JEOL model JEM-2100F instrument operated at 200 kV. Fourier transform infrared spectra (FTIR) was collected using KBr pellets by a Biorad FTS-60A equipped with a diffuse reflectance unit. Ultraviolet-visible (UV-vis) absorption spectra was performed using a spectrophotometer (UV-2500, Shimadzu, Japan), and BaSO_4_ as the reference was adopted. X-ray photoelectron spectroscopy (XPS) analysis was conducted using a Thermo ESCALAB 250 spectrometer with a hemisphere detector at an energy resolution of 0.1 eV offered by an Al Ka radiation source. The photoluminescence spectra (PL) was examined with a fluorescence spectrophotometer (F-4600, Hitachi, Japan). The Brunauer-Emmett-Teller (BET) specific surface areas of the sample powders were obtained through nitrogen adsorption-desorption, measured on an ASAP 2020 micromeritics surface and porosity analyser (USA). The visible light was supplied by a 300 W Xe arc lamp (PLS-SXE 300, Beijing Perfect Light Co., Ltd.) with a cutoff filter (λ > 400 nm) as the light source.

### The photocatalytic and photoelectrochemical performance test of the materials

The photocatalytic activities of the catalysts were detected through the degradation of Rh B operated by a 300 W Xe arc lamp equipped with a cut-off filter (λ > 400 nm) for visible light. For each experiment, 20 mg of photocatalyst was added into 60 mL Rh B solution (10 mg L^−1^). Before illumination, the suspensions were stirred in dark for 30 minutes to reach the adsorption-desorption equilibrium. Afterwards, the Rh B solution was recovered to initial concentration by exchanging 1.0 mg/L Rh B solution into the suspensions. At given time intervals, 4.0 mL suspension was withdrawn and centrifuged to remove the photocatalyst particles and then was tested by a UV-vis spectrophotometer to determine the concentration of Rh B through observing the 554 nm absorption peak. The degradation ratio was defined as (C_0_-C)/C_0_, where C referred to the absorption of dye in solution at a certain moment and C_0_ referred to the absorption of the initial concentration at each time point.

The photoelectrochemical analysis was conducted by Transient photocurrent response and Electrochemical impedance spectroscopy (EIS) measurements in a three-electrode system on a CHI650D electrochemical work station, using the BWO, BWO/G, BWO/M and BWO/MG powders deposited onto conducting glass (fluorine-doped tin oxide (FTO) substrate) with a working area of 1.0 cm^2^ as the working electrode, saturated calomel electrode (SCE) as the reference electrode, Pt wire as the counter electrode and Na_2_SO_4_ (0.5 M) as electrolyte. A 300 W Xe lamp with a 400 nm cut-off filter was used for excitation with external 0.5 V.

## Electronic supplementary material

Supplementary Information
