# Supplementary material for: Improving Photocatalytic Performance from Bi2WO6@MoS2/graphene Hybrids via Gradual Charge Transferred Pathway
Source: Sci Rep. 2017 Jun 16;7:3637. doi: 10.1038/s41598-017-03911-6 (PMC5473864; doi:10.1038/s41598-017-03911-6)
Supplement: Supplementary file 1 — Supplementary Information [file 41598_2017_3911_MOESM1_ESM.pdf]

Improving Photocatalytic Performance from $Bi_2WO_6@MoS_2$/Graphene Hybrids via Gradual Charge Transferred Pathway

Ming Liu[1], Xin Xue[2], Shansheng Yu[1], Xiaoyi Wang[5], Xiaoying Hu[4], Hongwei Tian[1,*], Hong Chen[3], Weitao Zheng[1,3]

[1] Department of Materials Science and Key Laboratory of Automobile Materials of MOE, Jilin University, Changchun, China.

[2] The Second Hospital, Jilin University, Changchun, China

[3] State Key Laboratory of Automotive Simulation and Control, Jilin University, Changchun, China.

[4] College of Science, Changchun University, Changchun, China

[5] Key Laboratory of Optical System Advanced Manufacturing Technology, Changchun Institute of Optics, Fine Mechanics and Physics, Chinese Academy of Sciences, Changchun, 130033, China

*Corresponding author: E-mail: tianhw@jlu.edu.cn.

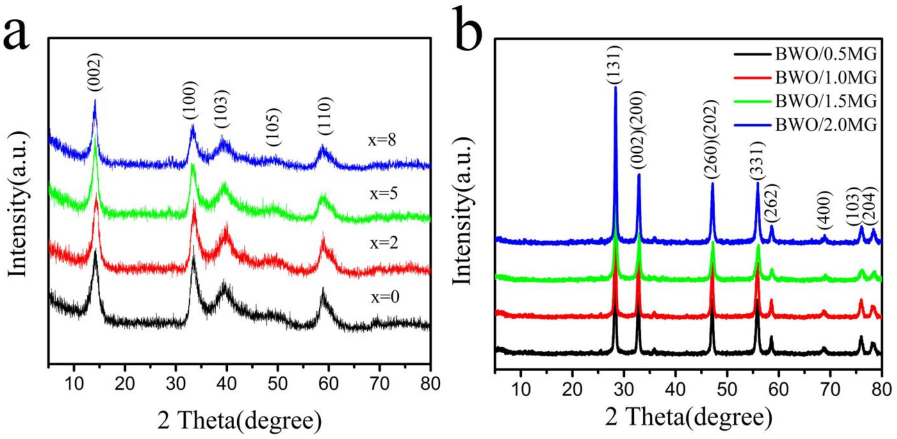

Figure S1. XRD patterns of (a) MoS_2/xG (x= 0, 2, 5, 8) and (b) pristine BWO/xMG (x= 0.5, 1.0, 1.5, 2.0).

As is shown in Fig. S1a, all the diffraction peaks of the hybrids are well indexed according to MoS_2 phase. In contrast to the BWO, it can be seen in Fig. S1b that the main diffraction peaks of BWO/xMG are similar to those of pure BWO. No obvious diffraction peak attributes to MoS_2 or graphene is observed, which suggests that low content and the stacking of the graphene sheets disordered.

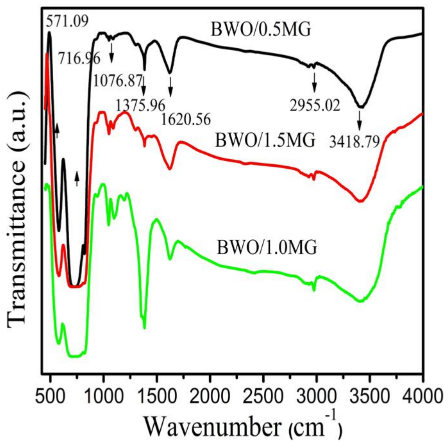

Figure S2. FTIR spectra of BWO/xMG (x= 0.5, 1.0, 1.5).

The FTIR spectra of BWO/MG with different mass ratio of cocatalsts are shown in Fig. S2. The representative absorption peaks of RGO of MG in BWO/MG including those at 3418.79 cm^{-1}, 1620.56 cm^{-1}, 1375.96 cm^{-1}, and 1076.87 cm^{-1}, are O-H stretching vibration, C=O stretching vibration of COOH groups, tertiary C-OH stretching vibration and C-O stretching vibration in good agreement with previous work, respectively. BWO has main absorption peaks between 500 and 1200 cm^{-1}, which can be attributed to stretching vibration of Bi-O and W-O, and bending vibration of W-O-W. Obvious decreases in intensity in FTIR spectrum of BWO/MG indicate that the oxygen-containing functional groups in MG are decomposed in the hydrothermal environment. The absorption band appearing at 1620.56 cm^{-1} clearly shows the skeletal vibration of the graphene sheets, indicating the formation of MG in BWO/MG.

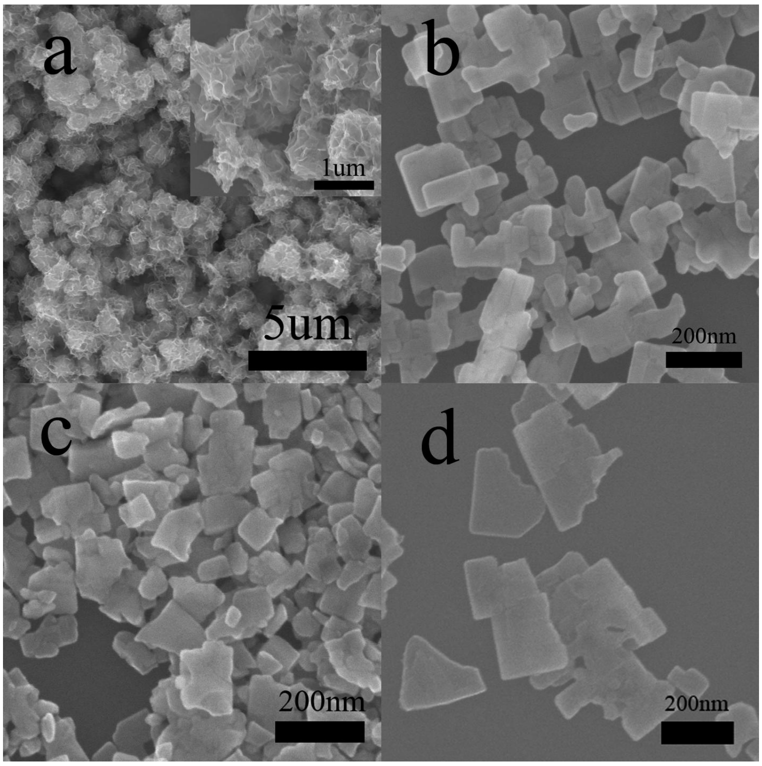

Figure S3. SEM images of (a) MG and (b-d) BWO/xMG (x= 0.5, 1.5, 2).

Fig. S3a shows that the MoS_2 and graphene in the hybrid samples are in a very close contact. In Fig. S3b-d, the SEM images of the different mass ratio ternary composites show that the average size of the nanosheets was 100-200nm, 100-200nm and 300-500nm corresponding to BWO/0.5MG, BWO/1.5MG and BWO/2.0MG, respectively.

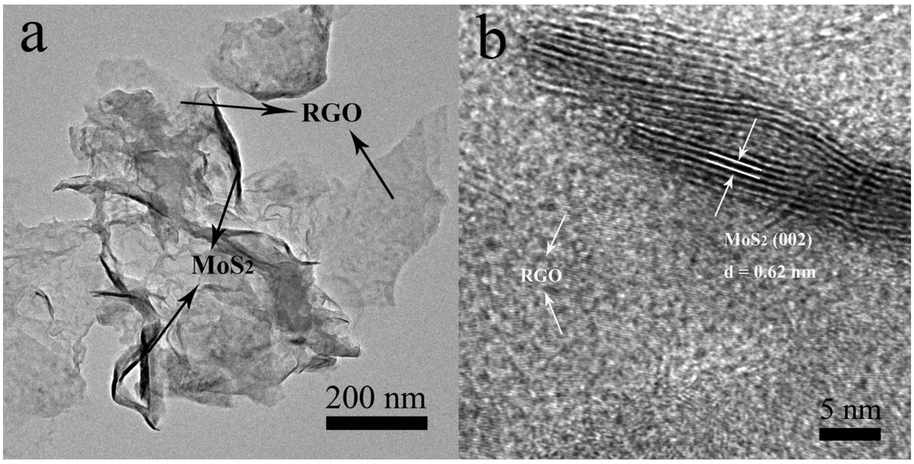

Figure S4. (a) TEM image and (b) HRTEM image of MG.

Graphene can be clearly observed in the binary MG cocatalyst, as shown in Fig. S4. MG composite has a layered structure with interlayer spacing of ca. 0.62nm which corresponds to the (002) planes of hexagonal MoS_2.

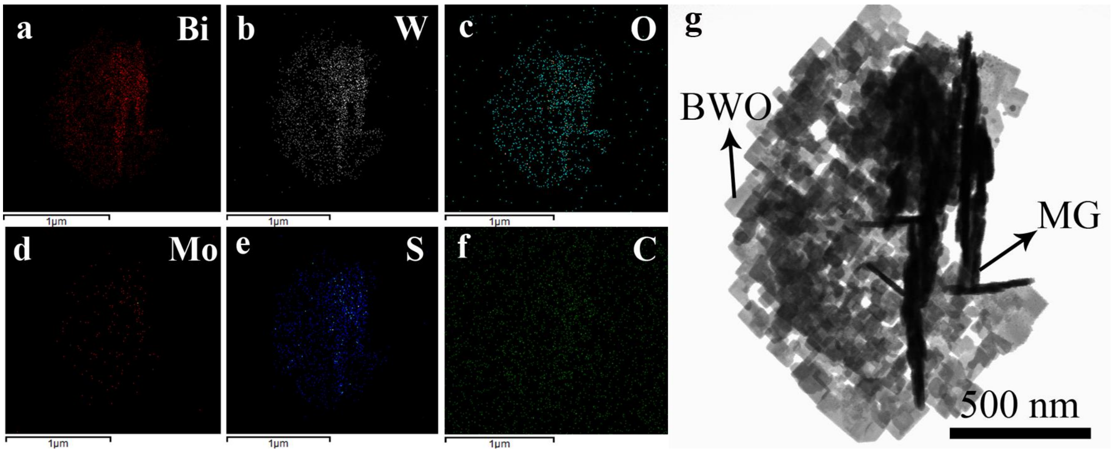

Figure S5. (a-f) The elemental mapping patterns of (g) the selected area for BWO/MG.

Based on the selected region in Fig. S5g, we can see the different-color elements in the mapping images for Bi, W, O, Mo, S and C elements in Fig. S5a-f, indicating that MG nanoparticles were successfully deposited on the surface of BWO afterhydrothermal procedure. After two-step hydrothermal procedure the content of graphene is only 0.05% (5%×1%) in the ternary heterojunction BWO/MG, though which is very little, but also can be observed in Fig. S5f.

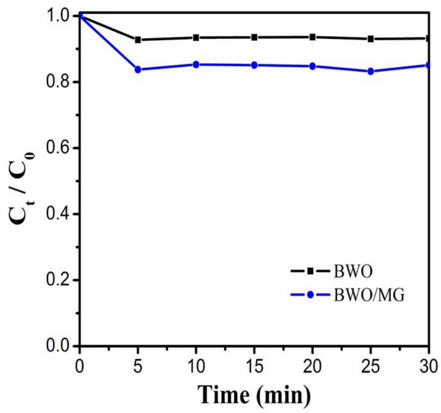

Figure S6. The adsorption-desorption equilibrium of BWO and BWO/MG for degrading Rh B in dark.

Before illumination, the suspensions were stirred in dark for 30 minutes to reach the adsorption-desorption equilibrium, as shown in Fig. S6.

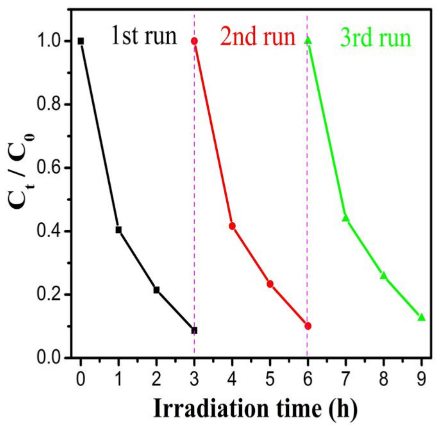

Figure S7. The recycling runs of BWO/MG for degrading Rh B under visible light.

As shown in Fig. S7, the degradation efficiency shows a slight decrease after three recycling runs, which directly certify the chemical stability of BWO/MG in the Rh B photodegradation process under visible light irradiation.

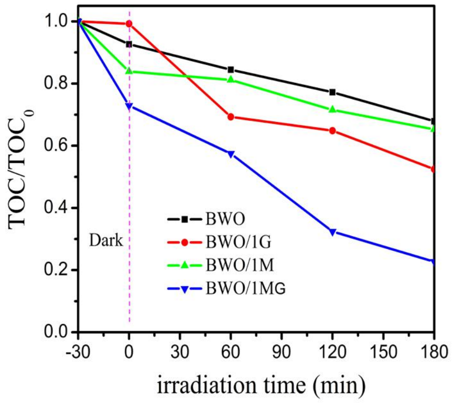

Figure S8. TOC removal of the Rh B for BWO and composite photocatalysts with 1% cocatalysts. Reaction conditions: Rh B concentration, 10 mg/L; the mass of catalyst, 20 mg; under visible light irradiation.

The photocatalytic performance of BWO, BWO/1G, BWO/1M and BWO/1MG (contains 95% of MoS_2 and 5% of graphene in the cocatalyst MG) in aqueous contaminant can also be evaluated by TOC removal. The change of TOC concentration reflected the mineralization degree of Rh B dye in Fig. S8. The TOC removal contents of BWO, BWO/1G, BWO/1M and BWO/1MG, respectively, are 32.77%, 34.73%, 47.55% and 77.31% under the visible light irradiation for 180 min. The ternary BWO/1MG catalyst shows the highest TOC removal rate of Rh B among the tested samples, which basically coincides with the result of the degradation rate. This phenomenon suggests that Rh B molecules are most likely mineralized into inorganic molecules.

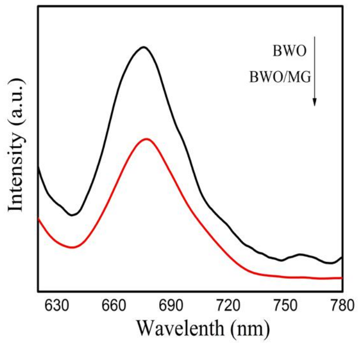

Figure S9. PL spectra of pure BWO and BWO/MG under 514nm excitation wavelength.

Fig. S9 demonstrates the PL spectra for pure BWO and BWO/MG samples under 514nm excitation wavelengths. The samples present a strong emission peak around 679nm in Fig. S9, owing to the intrinsic luminescence of samples. It is found that the PL intensity of BWO/MG is lower than that of pure BWO, which clearly indicates the inhibition of the recombination of excited electrons and holes in BWO/MG catalysts.

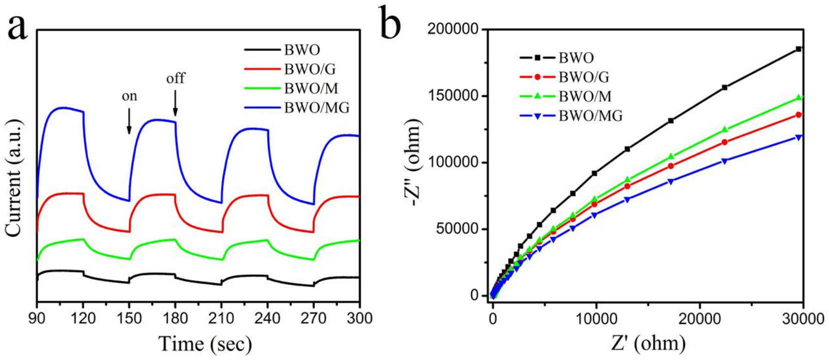

Figure S10. (a) Transient photocurrent response and (b) electrochemical impedance spectroscopy (EIS) Nyquist plots of the sample electrodes of the pure BWO, BWO/G, BWO/M and BWO/MG treated with visible light (λ>400 nm).

For attesting to the favourable effect of the graphene and MoS_2 in BWO/MG toward the light harvesting and transfer of photoexcited electron-hole pairs, photoelectrochemical analysis was performed under visible light. As shown in Fig. S10a, the transient photocurrent responses of BWO, BWO/G, BWO/M and BWO/MG photoelectrodes were still reproducible after several on-off cycles of regular irradiation of the visible light. The BWO/MG photo-electrode had the markedly highest photocurrent responses compared to BWO, BWO/G and BWO/M, which hints at the more efficient transmission and the longer-time separation for the photogenerated carries. The better conductivity of BWO/MG can be confirmed by the Electrochemical impedance spectroscopy (Fig. S10b). BWO/MG electrode has a smaller frequency semicircle compared with BWO, BWO/G and BWO/M electrode which meanings the resistance of BWO/MG electrode is lower with the best electrical conductivity, would best facilitate the migration of the photoexcited carriers, and therefore, the photocatalytic efficiency can be enhanced.

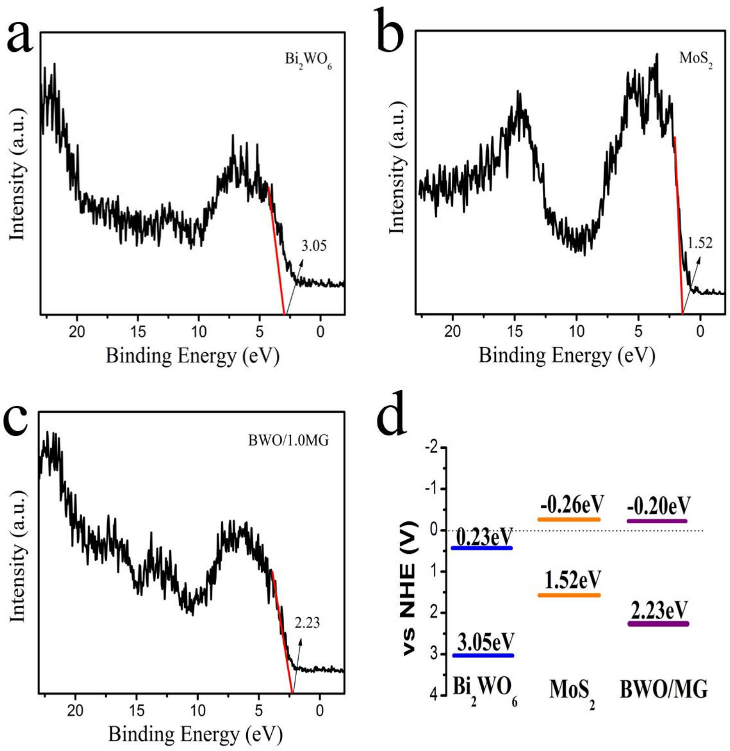

Figure S11. VB XPS spectra of (a) BWO, (b) MoS_2 and (c) BWO/MG. (d) Schematic illustration of the DOS of BWO, MoS_2 and BWO/MG.

The density of states (DOS) of the VB was obtained in VB XPS spectra to confirm the energy band structures of MoS_2 and BWO. The DOS of the VB is presented in the Fig. S11. The BWO displayed VB DOS with the edge of the maximum energy at about 3.05 eV. According to the UV-vis, the band gap of BWO is 2.82 eV. Thus, the CB minimum would present at about 0.23 eV by calculation. The DOS of VB in the MoS_2 with the edge of the maximum energy at about 1.52 eV, the CB are calculated to be about -0.26 eV. From the DOS of valence bands (VBs) of these samples, the VBs of BWO have changed after MG modification (2.23 eV). The schematic illustration of the DOS of BWO, MoS_2 and BWO/MG is shown in Fig. S11d.

Sample[a]	G[b](wt %)	S_{BET}[c](m^2/g)	APS[d](nm)	Vp[e](cm^3/g)	1-C/C_0 [f](%)	K
BWO/100M0G	0	6.701	9.225	0.077	65.07	0.00502
BWO/98M2G	2	7.408	7.798	0.070	74.19	0.00680
BWO/95M5G	5	18.781	11.565	0.140	91.29	0.01151
BWO/92M8G	8	9.707	8.131	0.081	47.84	0.00310
BWO/0M100G	100	12.175	6.764	0.075	80.97	0.00799

Table S1. Effects of MoS_2 and graphene content in the MG hybrid cocatalyst on the BET properties and photodegradation rate of the BWO/MG composites studied.

[a] All composite photocatalyst samples contain 99% of BWO and 1% of co-catalyst in the composite photocatalyst.

[b] G denotes graphene content in the MoS_2/graphene hybrid co-catalyst.
[c] S_{BET} denotes specific surface area.
[d] APS denotes average pore size.
[e] Vp denotes pore volume.
[f] 1-C/C_0, photodegradation rate of the composite photocatalyst samples.

Sample[a]	MG[b](wt %)	S_{BET}[c](m^2/g)	APS[d](nm)	Vp[e](cm^3/g)	1-C/C_0 [f](%)	K
BWO/0MG	0	11.544	8.800	0.096	21.79	0.00118
BWO/0.5MG	0.5	22.523	8.575	0.087	77.38	0.00680
BWO/1.0MG	1.0	18.781	11.565	0.140	91.29	0.01151
BWO/1.5MG	1.5	18.502	7.483	0.081	40.55	0.00310
BWO/100MG	100	57.639	11.125	0.310	2.08	----

Table S2. Effects of MG hybrid co-catalyst content in the BWO/MG photocatalyst on the BET properties and photodegradation rate of the BWO/MG composites.

[a] All composite photocatalyst samples contain 95% of MoS_2 and 5% of G in the cocatalyst MG.
[b] MG denotes MG content in the composite photocatalyst.
[c] S_{BET} denotes specific surface area.
[d] APS denotes average pore size.
[e] Vp denotes pore volume.
[f] 1-C/C_0, photodegradation rate of the composite photocatalyst samples.
